# Breaking continuous flash suppression: competing for consciousness on the pre-semantic battlefield

**DOI:** 10.3389/fpsyg.2014.00460

**Published:** 2014-05-23

**Authors:** Surya Gayet, Stefan Van der Stigchel, Chris L. E. Paffen

**Affiliations:** Experimental Psychology, Utrecht UniversityUtrecht, Netherlands

**Keywords:** continuous flash suppression, visual awareness, consciousness, binocular rivalry, interocular competition, interocular suppression

## Abstract

Traditionally, interocular suppression is believed to disrupt high-level (i.e., semantic or conceptual) processing of the suppressed visual input. The development of a new experimental paradigm, breaking continuous flash suppression (b-CFS), has caused a resurgence of studies demonstrating high-level processing of visual information in the absence of visual awareness. In this method the time it takes for interocularly suppressed stimuli to breach the threshold of visibility, is regarded as a measure of access to awareness. The aim of the current review is twofold. First, we provide an overview of the literature using this b-CFS method, while making a distinction between two types of studies: those in which suppression durations are compared between different stimulus classes (such as upright faces versus inverted faces), and those in which suppression durations are compared for stimuli that either match or mismatch concurrently available information (such as a colored target that either matches or mismatches a color retained in working memory). Second, we aim at dissociating high-level processing from low-level (i.e., crude visual) processing of the suppressed stimuli. For this purpose, we include a thorough review of the control conditions that are used in these experiments. Additionally, we provide recommendations for proper control conditions that we deem crucial for disentangling high-level from low-level effects. Based on this review, we argue that crude visual processing suffices for explaining differences in breakthrough times reported using b-CFS. As such, we conclude that there is as yet no reason to assume that interocularly suppressed stimuli receive full semantic analysis.

## INTRODUCTION

### INTEROCULAR COMPETITION

When different images are presented to both eyes, observers tend to perceive only one of these images, whereas the other one does not give rise to a conscious percept (e.g., binocular rivalry, Alais and Blake, 2005; flash suppression, Wolfe, 1984; continuous flash suppression, Tsuchiya and Koch, 2005). Under certain conditions the suppressed image has the potency to affect behavior, but this depends on the required level of processing (for a review see, Lin and He, 2009). For instance, the potency of low-level image properties, such as spatial frequency (Blake and Fox, 1974; Blake et al., 2006), motion direction (Wade and Wenderoth, 1978; O’Shea and Crassini, 1981; Blake et al., 1999), color (White et al., 1978), and orientation (Wade and Wenderoth, 1978) to elicit behavioral adaptation effects is relatively unaffected by interocular suppression. Conceptual or semantic processing, however, is traditionally believed to be abolished for interocularly suppressed stimuli (e.g., Zimba and Blake, 1983; Cave et al., 1998; Blake and Logothetis, 2002; Dehaene et al., 2006; Kang et al., 2011). In general, the extent to which neural activity reflects interocularly suppressed stimulation decreases gradually when climbing up the visual hierarchy (Blake and Logothetis, 2002). For instance, most cells in early visual areas (80% in V1/V2 and 60% in V4/V5) respond to stimulation of either eye irrespective of the dominant percept (Logothetis, 1998). Higher processing areas such as IT, FFA, and PPA, however, follow mostly (although not exclusively; Fang and He, 2005; Jiang and He, 2006; Sterzer et al., 2008) the dominant percept (Tong et al., 1998). Thus, interocularly suppressed stimuli are expected to be processed at the level of features and coarse feature configurations, which we will refer to as the lower or visual processing level, but not at a semantic or conceptual level (Blake and Logothetis, 2002), which we will refer to as higher level.

In contrast to this traditional view, studies using a novel paradigm called breaking continuous flash suppression (b-CFS; Jiang et al., 2007) seem to demonstrate that high-level processing of interocularly suppressed stimuli can occur prior to conscious experience. In the present article we aim to demonstrate that the seemingly high-level effects obtained in these b-CFS studies can be accounted for by coarse visual processing of the stimuli under continuous flash suppression (CFS). For this purpose, we provide a complete overview of all studies up to date (30) using b-CFS. Additionally, we suggest a number of improvements to the b-CFS method that help dissociate competition at relatively high levels of processing (i.e., at a conceptual or semantic level) from competition at lower levels of processing (i.e., at a featural level, where color, orientation, etc. are processed).

### BREAKING CONTINUOUS FLASH SUPPRESSION

In the b-CFS paradigm, a high contrast dynamic pattern mask is presented to one eye, thereby effectively suppressing a stimulus of increasing intensity presented to the other eye. Eventually, the ocular dominance will reverse, such that the previously suppressed stimulus becomes visible. The time it takes for observers to detect the suppressed stimulus is assumed to reflect the moment in time at which the stimulus gains access to consciousness. Importantly, non-ocular factors can affect the moment at which interocularly suppressed stimuli become consciously observable (Blake, 2001; Paffen and Alais, 2011). In light of the b-CFS paradigm, we dissociate two factors that co-determine the timing of an ocular dominance reversal. First, some stimulus classes might inherently breach the threshold of visibility faster than other stimulus classes (e.g., upright versus inverted faces; Jiang et al., 2007). Second, suppression durations can systematically differ for stimuli that either match or mismatch consciously accessible information (e.g., prime-target congruency; Costello et al., 2009). In reviewing the b-CFS literature we propose to take into account these two distinct ways in which non-ocular factors impinge upon the selection for conscious access: manipulations of the content of the suppressed stimulus, and manipulations of the context within which the suppressed stimulus is presented. As both types of experiments have their own advantages and limitations in uncovering the nature of preconscious processes, they are discussed separately.

## EFFECTS OF STIMULUS CONTEXT

### PRIMING

The first part of this review comprises an overview of b-CFS studies in which the detection time of identical stimuli is compared between different experimental conditions. These studies show that the same visual input can result in different suppression durations depending on the (consciously accessible) context that is provided. One widely studied way to affect the context within which information is presented is priming. This method involves presenting a stimulus prior to the b-CFS task, which is either related or unrelated to the masked target stimulus. Costello et al. (2009) showed that written words (e.g., “fire”) break through suppression faster when they are preceded by a word that shares sub-word components (e.g., “tire”) than when they are preceded by a word that does not share sub-word components. Costello et al. (2009) also showed that words break through suppression faster when they are preceded by a semantically related word (e.g., “burn”) than when they are preceded by an unrelated word. Lupyan and Ward (2013) took this one step further by showing that this priming effect also occurs when prime and target are presented in different modalities; for instance, an image of a pumpkin broke through suppression faster after observers heard the word “pumpkin” than after hearing a word that did not match with the subsequent target. Yang and Yeh (2014) presented words under CFS, of which the onset was either accompanied by an audible white noise burst or not. Detection times were shortened by the concurrent presentation of noise bursts, but only when the audio and visual information originated from the same depth plane. Together, these priming studies reveal that visual input that matches previously perceived information breaks through suppression faster than visual input that mismatches this information. Importantly, the prime-target relation can be spatial, physical, or semantic in nature, and does not require presentation in the same modality.

### THE CONTENT OF VISUAL WORKING MEMORY

Similarly to priming, the content of visual working memory is also known to affect visual processing, such that stimuli matching this content receive privileged processing compared to non-matching information (e.g., in search tasks, Olivers et al., 2006). One major difference between these two methods is that visual working memory involves the active, rather than passive maintenance (i.e., rehearsal) of visual features. In experiments that manipulate the content of visual working memory, participants are instructed to retain some feature of a visual stimulus for subsequent recollection. During the retention phase, participants perform a b-CFS task in which interocularly suppressed targets either match or mismatch the information that is concurrently retained in working memory. Recently, it has been shown that target stimuli under CFS are detected faster when they match rather than mismatch a color category (Gayet et al., 2013), an orientation (Liu et al., 2013) or a face (Pan et al., 2013) that is actively held in visual working memory. Crucially, detection times remain unaffected when the stimuli, otherwise used for the memory task, are passively viewed, as opposed to actively retained in working memory. In contrast with the priming studies discussed previously, Gayet et al. (2013) demonstrated that privileged detection of matching stimuli was only observed when the relevant stimulus dimension was retained; when participants retained the shape of a stimulus, targets that matched the color of that stimulus were not prioritized for conscious detection. Together, these working memory studies show that visual input that matches concurrently retained, task relevant information is accessible to consciousness faster than non-matching information.

### SIMULTANEOUS CROSS MODAL PRIMING

Three recent studies used a methodological approach in which the manipulation of the context was longer lasting than that of priming studies, without involving the active retention of information as in the working memory studies. In these experiments, consciously accessible, non-visual information was concurrently presented with a b-CFS task. First, Zhou et al. (2010) demonstrated that images matching olfactory information (e.g., an image of a rose concurrently presented with the scent of a rose) break through suppression faster than images mismatching olfactory information (e.g., an image of a rose concurrently presented with the scent of butanol). Second, Alsius and Munhall (2013) showed that an interocularly suppressed talking face stimulus broke through suppression faster when an auditory sentence matched rather than mismatched the lip synchronization of the face. Finally, Salomon et al. (2013) showed an effect of proprioception on visual awareness. In their study, participants reported the orientation of an interocularly suppressed target, which was superimposed on a task-irrelevant image of a hand. This hand could either be congruent or incongruent with the participants’ actual position. Targets broke through interocular suppression faster when the image of the hand matched the position of the real hand. The authors conclude that proprioception modulates the selection for conscious access of visual stimuli. Taken together, these studies show an advantage for detecting stimuli that match rather than mismatch consciously accessible information.

### VISUAL VERSUS CONCEPTUAL ANALYSIS OF SUPPRESSED STIMULI

The major advantage of all b-CFS experiments described in Sections “Priming to Simultaneous Cross Modal Priming is that differences in suppression durations cannot be accounted for by differences in image characteristics between conditions. This follows from the fact that in all conditions the same stimuli are used as target stimuli under CFS. The differentiation between conditions stems purely from the relation between target stimuli and the consciously accessible context in which they are embedded. Arguably, this context biases the competition by boosting or diminishing the effective strength of the suppressed stimuli (for a similar interpretation for attention’s effect on interocular suppression, see Paffen and Alais, 2011). The authors of the papers described above generally interpret their findings in terms of pre-activation of prime related information (either semantic or physical), which biases subsequent interocular competition (e.g., Lupyan and Ward, 2013). In this view, prime induced activity in areas further up the processing hierarchy (e.g., object selective areas) feeds back to the earlier visual cortex where the interocular competition is resolved (e.g., Blake, 1989; Tong, 2001). Note, however, that this interpretation cannot provide a satisfactory account for the semantic priming effect of Costello et al. (2009), which requires semantic analysis of the prime as well as the suppressed target. This issue is further discussed in Section “Assessing the Level of Processing.”

The assumption that competition occurred at the level of simple stimulus features rather than at the semantic or conceptual level was explicitly tested by Lupyan and Ward (2013) in a second experiment. Here, participants were cued with either the word “square” or “circle,” before performing a b-CFS task. By using a wide range of stimulus shapes ranging on a continuum from square to circle, they found that the similarity between the target stimulus and the cued shape was negatively correlated with the detection time of the target stimulus. The authors conclude from this finding that upon hearing (or reading) a word, a visual representation of its content is automatically activated. This active representation then facilitates subsequent detection of matching visual input. As such, the effects of semantic primes on suppression durations of subsequently presented targets are visual, rather than semantic in nature. The major advantage of this interpretation is that it allows for semantic priming, in the absence of semantic analysis of the suppressed stimulus.

Further support for this idea of feature pre-activation comes from the working memory experiments described above. When observers actively retain stimulus features, such as an orientation, these features can be decoded from activity in the early visual cortex (Harrison and Tong, 2009; Serences et al., 2009; Christophel et al., 2012). Thus, the abovementioned working memory studies allow for comparing between the situation in which prime-induced activity is retained and conditions in which prime-induced activity is discarded. The absence of an effect of the prime on suppression durations when the prime is perceived but not actively retained suggests that the prime-target congruency effects are indeed caused by pre-activation of prime induced features.

Together, the findings in this chapter show that providing a consciously accessible context prioritizes visual information that matches this context. As argued earlier, the consciously accessible context might activate a visual representation, which then interacts with the interocularly suppressed target. As such, even if the relation between the context and the suppressed target is semantic in nature, semantic analysis of the target is not *necessary* for detection times to be affected. One of the drawbacks of this type of b-CFS experiment is, however, that it does not allow for unequivocally excluding the possibility that the interocularly suppressed stimulus is processed up to a semantic level. In contrast, when comparing the potency of different stimulus classes in reaching visual awareness without providing a context, any difference in detection times between conditions (either featural or semantic in nature) reflects differences in the processing of the suppressed stimulus itself, rather than its interaction with a previously altered neural state. Studies using this approach will be discussed in the following paragraphs.

## EFFECTS OF STIMULUS CONTENT

### VISUAL CHARACTERISTICS

The second type of b-CFS experiments compares detection times between different stimulus categories. This comprises the comparison of stimulus categories that differ on the basis of relatively low-level visual properties that can be resolved in the early visual cortex, which will be discussed in this first section. For these stimulus properties, there is a tendency that more conspicuous stimuli are harder to suppress by CFS and, as such, break through suppression faster than less conspicuous stimuli. For instance, both higher contrast stimuli (Tsuchiya and Koch, 2005) and higher spatial frequency stimuli break through CFS more readily (Tsuchiya and Koch, 2005; Yang and Blake, 2012). Also, certain topological properties of interocularly suppressed stimuli elicit faster detection times than others. For instance, suppressed stimuli with a hole are detected faster than open stimuli made up of the same structural elements (Meng et al., 2012). When identical stimuli follow different motion patterns, this may result in different detection thresholds as well. For instance, coherently moving dot arrays break through suppression more often than random dot arrays that are presented for the same duration (Kaunitz et al., 2013). Climbing further up the visual hierarchy, images with strong grouping cues, such as Kanisza triangles are detected faster than non-Kanisza’s made up of the same constituents (Wang et al., 2012). Together, these studies show that different stimuli yield different suppression durations, and that this effect might be linked to the saliency of the suppressed stimulus. This is in line with findings from binocular rivalry experiments, which demonstrate that the location at which a perceptual transition is initiated depends on the local saliency of the suppressed stimulus (Paffen et al., 2008; Stuit et al., 2010).

Differences in suppression durations between stimulus categories can be accounted for both by properties of the suppressed stimuli *per se*, and by interactions between properties of the stimuli and properties of the masks (for a discussion, see Stein et al., 2011a). We dissociate two types of interactions between the stimuli and the masks that can potentially affect suppression durations. First, increased differences between visual characteristics of the suppressed image and the CFS stimuli reduce the suppression strength. For instance, Yang and Blake (2012) showed that stimuli with oblique orientations broke through suppression faster than stimuli with cardinal orientations, when using traditional “Mondrians” as CFS stimulus (which contain only cardinal orientations). More specifically, greater similarity in spatial frequency content and orientation between the competing percepts led to stronger suppression in both b-CFS (Yang and Blake, 2012) and binocular rivalry (Stuit et al., 2011). Second, when the previously suppressed image (or a sub-part of it) breaks through suppression, detection is facilitated if the suppressed image and the masks are very different. As discerning a suppressed stimulus through a mask requires exceeding some threshold of certainty, stimuli with more “proof” of being a potential target have an advantage in breaking CFS (for similar interpretations, see Kaunitz et al., 2013 and Yang and Yeh, 2014). Such a bias could be underpinned by the phenomenon of piecemeal rivalry, which allows for perceiving local parts of the “suppressed” stimulus (Blake et al., 1992; O’Shea et al., 1997). Since the dominant percept is highly dynamic (i.e., the CFS masks), locally dominant stimulus parts from the non-dominant eye (in which the target is presented) are easily confused with the CFS masks, and thus disregarded. However, when piecemeal rivalry reveals stimulus parts that seem coherently related (e.g., they follow a particular pattern or movement direction), these stimulus parts may attract attention, as they are likely to be the target (e.g., collinear facilitation; Wilson et al., 2001). This may affect suppression durations, since attending to a stimulus in a specific eye enhances the competition strength of the entire ipsi-ocular stimulus (Ooi and He, 1999; Zhang et al., 2012).

Nonetheless, differences between aforementioned conditions do not necessitate non-conscious semantic or conceptual processing, but are based on the differentiation of stimulus properties that are generally assumed to survive interocular suppression (for reviews, see Blake and Logothetis, 2002; Lin and He, 2009; Faivre et al., 2014; Sterzer et al., 2014). In the next sections, a number of studies will be discussed in which suppression durations are affected on the basis of higher level stimulus properties (i.e., at a semantic or conceptual level). Please note that the “familiarity” and “ecological relevance” distinction, as provided below, aims at categorizing these studies based on topical similarities, rather than describing the mechanisms that drive their results.

### FAMILIARITY

Differences in detection times between stimulus categories can also arise on the basis of more high-level distinctions, such as stimulus familiarity. For instance, images of human bodies or body parts are detected faster when presented upright as compared to inverted (Stein et al., 2012). As the authors demonstrate that this latter effect was abolished when the images were distorted, the authors argue that the difference in detection times is accounted for by the greater familiarity of upright human bodies. Along the same lines, upright faces are detected faster than inverted faces (Jiang et al., 2007; Zhou et al., 2010; Stein et al., 2011a,b; Gray et al., 2013). Two of these studies (Stein et al., 2011b; Gray et al., 2013) also included a polarity inversion condition, demonstrating that detection times were fastest for normal faces (upright and normal polarity) and slowest for the most unusual face presentation condition (spatial inversion and inversed polarity), although the inversion effect was only marginally significant in the inversed polarity condition of Stein et al. (2011b). The finding that face inversion effects are dependent on (or additive to) manipulations of the contrast polarity, supports the idea that it is indeed familiarity that drives the priority for detecting upright faces. Importantly, however, Stein et al. (2011b) replicated these findings with configurations of three blobs representing two eyes and a mouth. This demonstrates that the privilege for detecting upright faces can be resolved by very crude visual processing.

Gobbini et al. (2013b) took the manipulation of stimulus familiarity even further by showing that interocularly suppressed familiar faces are detected faster than faces of strangers. A more subtle finding comes from a study showing that faces from the own racial in-group break through suppression faster than faces from the racial out-group (Stein, 2012). That same study showed that faces of the same age group as that of the observer break through suppression faster than faces of another age group. Importantly, the differences in suppression durations between image conditions were computed relative to that of inverted faces, such that they could not be attributed to differences in low-level image properties (see Control 1: Disrupting the Extraction of Meaning). Rather, the authors suggest that this effect is accounted for by the observer’s greater visual expertise with stimuli of the own-race and own-age stimulus classes.

This facilitatory effect for detecting visual input of higher familiarity is also found for stimuli that are more recently acquired in evolutionary time, such as written language. Indeed, words in a familiar alphabet are detected faster than words in an alphabet that is unfamiliar to the observer (Jiang et al., 2007). Similarly, Chinese characters are detected faster by Chinese readers compared to the same characters that have been inversed or scrambled (Yang and Yeh, 2011, 2014). Taken together, these studies show that visual input with higher stimulus familiarity is more readily detected than less familiar input. Arguably, extended experience with certain types of stimuli might facilitate subsequent detection. If so, the factor of familiarity might be the long term equivalent of the stimulus feature pre-activation as described in Section “Effects of Stimulus Context.”

### ECOLOGICAL RELEVANCE

A number of studies demonstrate differences in detection times for stimuli that differ on the basis of ecological relevance. For instance, observers show an advantage for detecting faces turned toward the observer compared to faces turned slightly away from the observer (Gobbini et al., 2013a). This difference was found to be independent of the gaze direction of the face. Similarly, faces with direct gaze break through interocular suppression faster than faces with averted gaze. This was found both for schematic faces (Chen and Yeh, 2012) and for face photographs (Stein et al., 2011c). This advantage for detecting faces with direct gaze could not be explained by (lower-level) effects of eye symmetry, as Stein et al. (2011c) included images of both frontal faces and laterally averted faces, such that gaze direction should be inferred by the particular combination of both face orientation and pupil position. However, the advantage in detecting stimuli with direct gaze over averted gaze persisted for inverted faces (Stein et al., 2011c; Chen and Yeh, 2012). Gaze direction in (visible) faces is more difficultly inferred from inverted faces compared to upright faces (e.g., Vecera and Johnson, 1995). Thus, the effect of gaze direction on detection times should be less prominent in the inverted condition than in the upright condition. The absence of this interaction between gaze direction and face inversion therefore hints toward the interpretation that crude configural differences between gaze conditions might play a causal role in eliciting these differences in detection times. For instance, Chen and Yeh (2012) propose that the specific conjunction of face curvature and pupil location is sufficient in eliciting shorter suppression durations. In line with this idea, they demonstrated in an additional experiment that the mere schematic depiction of eyes was sufficient in explaining the observed difference in detection times of full (schematic) faces.

Another ecologically potentially relevant distinction between stimulus categories is that of emotional versus non-emotional stimuli. For instance, fearful faces break through suppression faster than neutral faces (Yang et al., 2007; Gray et al., 2013; Stein et al., 2014) or happy faces (Yang et al., 2007; Tsuchiya et al., 2009; Gray et al., 2013), while happy (Yang et al., 2007) and angry faces (Gray et al., 2013) break through suppression *slower* than neutral faces. Interestingly, both types of emotional expressions break through suppression *faster* than neutral faces when schematic face images are used instead of face photographs (Stein and Sterzer, 2012). This contradiction suggests that it is not the analysis of the emotional valence *per se*, but rather the visual properties of the image that affected suppression durations in these studies. In line with this lower level account, the findings of Gray et al. (2013) persisted for inverted faces and for faces with inversed polarity, while the findings of Yang et al. (2007) persisted for inverted faces and for eyes-only images. Similarly, the findings of Stein and Sterzer (2012) were fully accounted for by the relative orientation of the mouth curvature and the face contour. Finally, the findings of Stein et al. (2014) depended solely on high spatial frequency information. Since subcortical (i.e., amygdala) processing of fearful faces relies predominantly on low spatial frequency information (e.g., LeDoux, 1998), this finding suggests that non-conscious processing of fearful faces is dependent on cortical processing. Patient SM, who has complete bilateral amygdala lesions and is unable to consciously discriminate between fearful and happy faces, showed the same advantages for detecting CFS-suppressed fearful faces over happy faces as controls did (Tsuchiya et al., 2009). As such, non-conscious discrimination between emotional faces seems to rely more on (cortical) extraction of characteristic visual features, than on the (subcortical) analysis of the emotional valence *per se*. Taken together, these studies show a tendency for ecologically relevant stimuli to break through interocular suppression faster than less ecologically relevant stimuli. However, most of these effects have been shown to rely on stimulus properties, or stimulus configurations, that can be dissociated on the basis of relatively crude visual processing. In sum, semantic, conceptual or emotional analysis of interocularly suppressed stimuli is not a *necessary* condition to account for the observed differences in detection times. Rather, the extraction of purely visual information seems to sufficient to explain most of the findings discussed so far.

### CLIMBING TOWARD THE SEMANTIC AND CONCEPTUAL LEVEL

As with the privilege for detecting familiar stimuli, the privilege for detecting emotional stimuli was not restricted to evolutionarily old visual input, such as faces, but was also demonstrated for words (Yang and Yeh, 2011). Interestingly, the results of this study revealed that both (Chinese) words that *describe* a negative emotion (e.g., “anger” or “fear”) and words that *induce* a negative emotion (e.g., “murder” or “abuse”) were detected *later* than neutral words. Taking this idea even further, Sklar et al. (2012) compared suppression durations of emotionally negative expressions to suppression durations of neutral expressions. Importantly, the words that formed these expressions had no intrinsic emotional valence (e.g., “eternal” and “rest”; “eternal rest”). Nonetheless, the expressions with a negative emotional valence broke through suppression faster than neutral expressions. Interestingly, these results are at odds with that of Yang and Yeh (2011). Still, both studies demonstrate effects that require semantic processing of the words before interocular competition is resolved.

Sklar et al. (2012) also demonstrated that combinations of (Hebrew) words that yield incoherent expressions (e.g., “she ironed coffee”) broke through suppression faster than coherent expressions (e.g., “she drank coffee”). Again, it is the semantic combination of words that determines whether an expression is coherent or incoherent, rather than the individual words themselves. This finding demonstrates that the meaning of words is indeed extracted and integrated non-consciously. Along the same lines, Mudrik et al. (2011) showed that scenes containing incongruent objects (e.g., Michael Jordan holding a watermelon) broke through suppression faster than the same scenes containing congruent objects (e.g., Michael Jordan holding a basketball). The authors stress that dissociating a coherent from an incoherent image requires the integration of an object in its semantic context; a process originally thought to require consciousness (e.g., Edelman and Tononi, 2000). In contrast with the familiarity effects discussed in Section “Familiarity,” the stimuli used in these last two experiments seem too complex for the differences in suppression durations to be accounted for by differences in visual experience between stimulus conditions. As such, these results imply full blown semantic analysis of interocularly suppressed stimuli.

## DISCUSSION

### ASSESSING THE LEVEL OF PROCESSING

Most findings in Section “Priming” up to Section “Ecological Relevance” can be explained by preconscious analysis of suppressed stimuli at relatively early stages of visual processing. Whether they are caused by pre-activation of primed features, by the saliency of a stimulus, or by the long time strengthening of visual representations of relevant feature configurations, these findings do not seem to require semantic or conceptual processing. In contrast, the findings discussed in the last section (see Climbing Towards the Semantic and Conceptual Level) as well as the semantic priming effect of Costello et al. (2009) seem to defy the model of early competition in interocular suppression and point to high-level analysis of the suppressed stimuli. There are, however, two reasons to plead for caution in interpreting the studies that demonstrate these high-level effects (e.g., language and scene comprehension). First, some of the results described above seem contradictory, such as the results of Sklar et al. (2012) in which negative emotional expressions yielded shorter suppression durations compared to the results of Yang and Yeh (2011) in which negative emotional words yielded longer suppression durations. In a broader sense, the overall pattern of findings of these high-level effects (see Climbing Towards the Semantic and Conceptual Level) seems inconsistent with the pattern of findings from lower level effects (Sections Priming – Ecological Relevance). On the one hand, words and images break through suppression faster when they have a higher prevalence in the observers’ visual world (i.e., when they are of higher familiarity). On the other hand, however, word combinations and complex scenes break through suppression faster when they are incongruent or novel, and thus are of lower familiarity. While it is conceivable that scene complexity influences the magnitude of the effect of familiarity on suppression durations, it is unexpected that scene complexity causes a reversal in the direction of the effect of familiarity on suppression durations.

Second, to demonstrate that differences in suppression durations are caused by competition at a high processing level (i.e., semantic or conceptual), it is important to implement a comparison with a condition that disrupts high-level processing, such as inversion (e.g., as used in the Sterzer lab), polarity inversion or scrambling. If the difference in suppression durations observed under normal presentation conditions is also apparent in these conditions, it is likely that the effect is caused by differences in lower level visual properties between the stimulus classes (see Control 1: Disrupting the Extraction of Meaning). Four out of five studies that do include this type of control conditions to dissociate between competition at higher processing levels from competition at lower (visual) processing levels, demonstrated that the effect could indeed be attributed to competition at lower levels of the processing hierarchy (Stein et al., 2011b; Stein and Sterzer, 2012; Chen and Yeh, 2012; Gray et al., 2013). Consequently, these studies do not attribute their findings to high level processing under continuous flash suppression. Importantly, three out of four b-CFS experiments that led the authors to conclude from their data that the observed difference in suppression durations was caused by semantic or conceptual analysis of the stimuli under CFS, however, did not include such a control condition (i.e., Costello et al., 2009; Mudrik et al., 2011; Sklar et al., 2012). Thus far, the only study that convincingly demonstrates high-level competition in a b-CFS experiment, is that of Yang and Yeh (2011). In this study, the authors included an inversion condition, a scrambled condition as well as a monocular condition. This revealed that the shorter suppression durations for neutral Chinese words compared to emotional Chinese words was only apparent in the upright unscrambled dichoptic condition.

In sum, more and more studies (discussed in Climbing Towards The Semantic and Conceptual Level) aim at demonstrating that semantic and conceptual information might be integrated non-consciously. However, this is hard to reconcile with studies showing that semantic priming effects are abolished under interocular suppression (e.g., Zimba and Blake, 1983; Cave et al., 1998; Kang et al., 2011; for reviews, see Lin and He, 2009; Faivre et al., 2014; Sterzer et al., 2014). In some studies interocular suppression is even used as a tool to disrupt semantic processing (e.g., Lupyan and Ward, 2013). These high-level effects are also hard to reconcile with the idea that interocular competition is resolved in early visual areas such as LGN (Haynes et al., 2005) and V1 (Polonsky et al., 2000). Although some interocularly suppressed information is known to transpire into higher visual areas (e.g., Fang and He, 2005; Jiang and He, 2006; Sterzer et al., 2008), succeeding levels in the processing hierarchy reveal less and less brain activity that reflects interocularly suppressed stimulation (Blake and Logothetis, 2002). Moreover, CFS is known to result in greater suppression depths than more traditional methods of interocular suppression, such as flash suppression and binocular rivalry (Tsuchiya et al., 2006). Consequently, when b-CFS is used to compare different classes of stimuli in their potency to breach the threshold of awareness, it is of utmost importance to test whether reaction times indeed reflect differences in high-level rather than low-level information in the stimuli. Additionally, irrespective of the processing level at which the competition takes place, it is crucial to assert whether reaction times indeed reflect differences in the timing at which a stimulus was available to consciousness, rather than processes arising after the stimulus became available to consciousness. These post-perceptual effects pose a threat to b-CFS experiments in which the stimulus content is manipulated as well as to experiments in which the stimulus context is manipulated. We propose that at least the following three control conditions should be included in b-CFS experiments to control for these potential pitfalls.

### CONTROL 1: DISRUPTING THE EXTRACTION OF MEANING

To assess whether differences between conditions rely on high-level information (i.e., at a semantic or conceptual level), one or more conditions should be included that are known to disrupt the extraction of high-level image properties, while keeping low-level (i.e., visual) image properties relatively unaffected. This can be achieved by such manipulations as inverting the image or inverting the image polarity (e.g., Jiang et al., 2007; Zhou et al., 2010; Stein et al., 2011b; Chen and Yeh, 2012; Gray et al., 2013). These manipulations constrain the extraction of meaning from an image (Rock, 1974; Shore and Klein, 2000), such that high-level driven effects should at least diminish under these circumstances. As such, if some image class breaks through suppression faster than another stimulus class because of high-level (i.e., semantic or conceptual) differences, the differences in detection times between these two stimulus classes should not be observed (or at least diminish) when the images are presented upside down. Conversely, if the difference in detection times between two stimulus classes does persist with inverted presentation, this suggests that there are systematic low-level visual differences between the two image classes, as these should remain unaffected by inverted presentation. In that case, the differences in low-level visual properties are the probable cause of the difference in detection times between the two stimulus classes. For this reason, rather than looking at absolute detection times for each stimulus category, it is more informative to look at the inversion effect, which is described as the difference in detection times between upright and inverted stimuli of the same stimulus category. This difference can then be divided by the detection time of inverted stimuli (as in Stein, 2012) such as to remove the between subject variability in detection times. Consequently, to assess whether a difference between image classes relies on high-level stimulus processing, it is important to demonstrate that the inversion effects (rather than the detection times *per se*) differ between stimulus classes.

### CONTROL 2: STIMULUS REPORTABILITY

Next, it is important to verify whether differences in reaction times indeed reflect differences in visual awareness. An alternative view is that differences in reaction times are driven by non-conscious processes, such that stimulus information is accessible only to the extent that it affects forced choice localization, while not being accessible to subjective report. Arguably, a stimulus fails to reach visual awareness, if it is accessible to one output system, but not to the other (Baars, 1993; Kanwisher, 2001). Thus, in order to conclude that some manipulation in a b-CFS experiment affects visual awareness, visual awareness should be measured directly. Visual awareness of a stimulus is assumed to be a prerequisite for stimulus reportability (Dehaene et al., 2006). As such, it can be operationalized as the ability to subjectively report ones percept (Dennett, 1993; Weiskrantz, 1997; Dehaene and Naccache, 2001). For the present purpose, a direct way to test whether one stimulus was accessible to consciousness and the other was not, is to compare participants’ ability to report the identity of two concurrent stimuli at a particular point in time. This objective measure of stimulus reportability can be implemented by presenting two stimuli of different conditions simultaneously (e.g., one at either side of fixation). After participants perform a speeded detection of the location at which (e.g., left or right of fixation) they first see a stimulus appear, they should report either the identity of the percept on the reported location, or of that on the non-reported location. If no post-detection strategic bias is involved, participants should be significantly worse at reporting the identity of the stimulus on the non-reported location as compared to that of the reported location. Conversely, if participants are equally proficient at reporting the identity of either stimulus, one may not conclude that there was a difference in conscious access between stimulus conditions.

### CONTROL 3: POST DETECTION EFFECTS

Finally, it is important to assess whether differences in detection times indeed reflect differences in interocular suppression durations, rather than processing differences arising after conscious detection of the stimulus (e.g., a difference in response criterion). To account for these “late” effects it is imperative to add a monocular (or binocular) control condition, in which the “suppressed” stimulus and the CFS are presented to the same eye(s). Specifically, we advocate the use of two different monocular control conditions (as in, Costello et al., 2009; and Gayet et al., 2013). First, a monocular control condition is needed in which the presentation times are identical to that of the interocular condition, such as to keep the stimulus chronology constant (i.e., a physically similar control). The disadvantage of this condition is, however, that reaction times in this condition are much faster than in the interocular condition. Consequently, any differences in reaction times between conditions are reduced in magnitude as well, as a result of which the experimental power can be diminished (although the variance is reduced as well). Thus, it is imperative to implement a second monocular condition such, that the reaction time distributions (means and SD’s) match that of the interocular condition (for further discussion on this issue, see Stein et al., 2011a). This can be achieved by (1) lengthening the ramp of the “suppressed” stimulus, such as to mimic the longer suppression durations of trials with dichoptic presentation, and, (2) by jittering the target onset, such as to add uncertainty as to when the target will appear (i.e., a perceptually similar control). Ideally, interocular trials and monocular control trials are randomly intermixed within blocks. This has the main advantage of making the perceptual difference between dichoptic and monocular (or binocular) presentation conditions less conspicuous, due to the whimsical nature of dichoptically presented trials.

Together, these three methods provide empirical tests for (1) whether differences between stimulus conditions actually rely on high-level information, (2) whether differences in reaction times indeed reflect differences in explicit visual awareness, and (3) whether reaction times were affected by processing differences emerging after conscious detection, such as changes in response criterion.

### INTERPRETING THE RESULTS OF b-CFS STUDIES

As mentioned in the introduction, the rationale underlying b-CFS experiments is that differences in suppression durations between conditions reflect different processing of stimuli prior to conscious access. An often disregarded alternative, however, is that differences between conditions may affect visual processing *during* the transitory period in which the interocularly suppressed stimuli gradually gain access to consciousness. In support of this latter idea, CFS allows for periods of partial awareness, in which some, but not all, features of a stimulus are suppressed (Zadbood et al., 2011; Yang and Blake, 2012). Crucially, Mudrik et al. (2013) demonstrated that “non-conscious” processing of faces was restricted to periods of partial awareness. This finding has two consequences for b-CFS studies: First, it indicates that detection tasks are better suited than discrimination tasks to ascertain that differences in detection times between conditions are initiated prior to a switch in ocular dominance. For instance, if the crucial manipulation involves one feature of some stimulus (e.g., color) and participants are required to report another feature of that stimulus (e.g., orientation) for the b-CFS task, it is conceivable that the color of that same stimulus was accessible to consciousness prior to its orientation. As a result, the possibility cannot be excluded that the process driving the differences in detection times between color conditions arose after the interocular competition (of that particular feature) was resolved, thereby reflecting a conscious rather than non-conscious process. Second, and more importantly, if the main goal of the experimenter is to uncover the nature of non-conscious processing, the concurrent usage of multiple suppression techniques is advisable, with an emphasis on the more traditional methods that have been ostensibly validated (Stein and Sterzer, 2014) and are less susceptible to partial awareness (Mudrik et al., 2013). As suggested above, b-CFS possibly relies on processing differences during the transitory period, in which previously suppressed stimuli gain gradual access to consciousness. As such, it is hard to ascertain whether differences in stimulus processing during a transition of ocular dominance can generalize to differences in stimulus processing in the complete absence of consciousness. Despite being not as well suited as a tool to uncover non-conscious processing *per se*, b-CFS experiments are nonetheless very informative as a measure of access to awareness (Stein and Sterzer, 2014). Consequently, the results of b-CFS experiments should be interpreted as such.

With the above mentioned additions to the b-CFS paradigm, we hope to provide the means to effectively dissociate situations in which competition for conscious access occurs on high-level battle grounds and thus requires conceptual or semantic processing, from situations in which the competition occurs on lower level battle grounds, such that crude visual processing of the suppressed stimuli suffices. In light of the abovementioned limitations, it should be emphasized that whether or not high-level stimulus properties exert influence on conscious access within the b-CFS paradigm does not necessarily imply that the same restrictions apply to non-conscious processing under CFS, let alone to interocular suppression in general. Thus far, however, the idea that interocularly suppressed stimuli are not analyzed up to semantic or conceptual processing levels has been mainly challenged by b-CFS experiments. The present review included 30 studies that use this experimental paradigm, of which 8 aimed to explore whether suppression durations could be affected by competition at a semantic or conceptual processing stage. Four of these studies demonstrate that these effects could be accounted for by differences in low-level visual properties, three of these studies did not include conditions that controls for differences in low-level visual properties, and as a result, only one study demonstrates high-level effects in a b-CFS task. As such, we conclude that interocular competition at a visual level is a sufficient explanation for most b-CFS studies that properly control for low-level visual differences (i.e., that include an inversion condition). As such, we should be reluctant to revise the traditional idea that semantic or conceptual analysis is abolished under interocular suppression.

## Conflict of Interest Statement

The authors declare that the research was conducted in the absence of any commercial or financial relationships that could be construed as a potential conflict of interest.
